# Evaluation of Austenite–Ferrite Phase Transformation in Carbon Steel Using Bayesian Optimized Cellular Automaton Simulation

**DOI:** 10.3390/ma16216922

**Published:** 2023-10-28

**Authors:** Fei Sun, Yoshihisa Mino, Toshio Ogawa, Ta-Te Chen, Yukinobu Natsume, Yoshitaka Adachi

**Affiliations:** 1 Department of Material Design Innovation Engineering, Nagoya University, Furo-cho, Chikusa-ku, Nagoya 464-8603, Japan; mino.yoshihisa.y7@s.mail.nagoya-u.ac.jp (Y.M.); chen.ta.te.w8@f.mail.nagoya-u.ac.jp (T.-T.C.); 2 Department of Mechanical Engineering, Aichi Institute of Technology, 1247 Yachigusa, Yakusa Cho, Toyota 470-0392, Japan; ogawa.toshio@aitech.ac.jp; 3 Department of Materials Science, Akita University, 1-1 Tegata-Gakuenmachi, Akita 010-8502, Japan; natsume@gipc.akita-u.ac.jp

**Keywords:** phase transformation, cellular automaton, Bayesian optimization, microstructure

## Abstract

Austenite–ferrite phase transformation is a crucial metallurgical tool to tailor the properties of steels required for particular applications. Extensive simulation and modeling studies have been conducted to evaluate the phase transformation behaviors; however, some fundamental physical parameters still need to be optimized for better understanding. In this study, the austenite–ferrite phase transformation was evaluated in carbon steels with three carbon concentrations during isothermal annealing at various temperatures using a developed cellular automaton simulation model combined with Bayesian optimization. The simulation results show that the incubation period for nucleation is an essential factor that needs to be considered during austenite–ferrite phase transformation simulation. The incubation period constant is mainly affected by carbon concentration and the optimized values have been obtained as 10^−24^, 10^−19^, and 10^−21^ corresponding to carbon concentrations of 0.2 wt%, 0.35 wt%, and 0.5 wt%, respectively. The average ferrite grain size after phase transformation completion could decrease with the decreasing initial austenite grain size. Some other parameters were also analyzed in detail. The developed cellular automaton simulation model combined with Bayesian optimization in this study could conduct an in-depth exploration of critical and optimal parameters and provide deeper insights into understanding the fundamental physical characteristics during austenite–ferrite phase transformation.

## 1. Introduction

The rapid advancement of data science in recent years has been widely recognized for its role in accelerating scientific progress as a whole. Within the realm of steel material development, data science has found application in both forward analysis, which predicts properties based on microstructures, and inverse analysis, which indicates microstructures and processes from properties [1]. An essential factor in ensuring reliable outcomes through data science is the availability of sufficient training data. Generally, the larger the dataset used for training, the more reliable the results obtained. However, a notable challenge arises because much of the training data used today are derived from experiments, making the expansion of training data a potential bottleneck. To overcome the challenge, two methods have been proposed to efficiently increase the volume of available data: “virtual generation of tissue images themselves” [2,3] and “modeling” [4]. An illustrative example of the former approach involves machine learning techniques like adversarial image generation networks. These methods have shown improvement in accuracy and efficiency while accompanying difficulties in establishing the physical underpinnings of the generated tissue images and challenges in evaluating the results. In contrast, the modeling method is distinguished by its capacity to efficiently generate data while maintaining a strong connection to the physical principles governing the phenomenon being studied. This physical foundation enhances our understanding of the underlying processes and makes modeling a valuable tool for predicting conditions across a wide range of scenarios.

Phase transformation is a critical phenomenon that significantly impacts the properties of steel [5,6,7,8]. The transformation from austenite to α-ferrite represents a crucial behavior in steels. It has been the subject of extensive investigation to explore the relationship between its microstructure and cooling conditions [9,10,11]. Austenite–ferrite phase transformation in steel is very important because the volume fraction and morphology of ferrite significantly affect the mechanical properties [12,13]. Extensive studies have been clearly performed to understand the austenite–ferrite phase transformation mechanisms, such as nucleation, kinetics, thermodynamics, and so on [14,15,16,17,18,19,20,21]. Various numerical modeling works have been also conducted to simulate the microstructure evolution and solute diffusion during the phase transformation. Microstructure modeling encompasses several commonly used and well-established methods, including the phase-field (PF) method, Monte Carlo (MC) method, and cellular automaton (CA) method [22]. The PF method is deterministic and has been extensively applied for micro-scale calculations, such as dendrite growth modeling [23]. After deformation, the PF model combined with the finite element method to present the austenite–ferrite phase transformation behavior [24]. In contrast, the MC method is inherently probabilistic and offers distinct advantages when dealing with macro-scale computations, with notable applications in predicting grain growth [25]. In addition, the MC method also simulated the austenite–ferrite phase transformation during the austenite decomposition during isothermal treatment [26]. The CA method occupies an intermediate position between these two approaches and is primarily employed for modeling the phase transformation mechanisms in the mesoscale [27,28,29,30,31,32,33,34,35,36,37,38]. Compared to the MC method, the CA method is deemed more reliable due to its stronger foundation in physical principles, which results in reduced stochastic elements. This method relies on a grid-based microstructure representation, where each cell can be in one of several discrete states corresponding to different phases or microstructural features. CA simulations allow researchers to model and analyze the complex processes involved in phase transformations, including nucleation, growth, diffusion, and crystallography, by applying rules that dictate how these cells evolve over discrete time steps. It could provide insights into how phase transformation occurs and affects the final microstructure and material properties. Notably, given that phase transformation involves stochastic and deterministic aspects, encompassing classical nucleation theory and atomic diffusion, the CA method is widely recognized as one of the most effective modeling techniques. Furthermore, the CA method’s ability to adequately address grain size considerations at the mesoscale is a distinct advantage in reproducing phase transformations.

Parameter optimization presents a significant challenge in enhancing modeling accuracy, and two well-known approaches to tackle this issue are data assimilation and Bayesian optimization. Data assimilation is primarily employed for estimating parameters in situations involving sequential changes and finds extensive application in fields like meteorology, particularly in forecasting phenomena such as typhoons and torrential rainfall. Bayesian optimization is utilized for parameter estimation in scenarios without a strict sequence of changes and is widely adopted in material texture modeling [39,40]. As a result, the primary objective of this study is to establish and refine modeling techniques that leverage the physical background for the advancement of steel material development through the combination of CA method and parameter optimization.

In this study, we focused on modeling the phase transformation of carbon steel using the CA method combined with Bayesian optimization techniques to explore and refine some critical parameters that have not been mentioned but need to be well considered during simulation, aiming to deepen the comprehension of the austenite–ferrite phase transformation. Moreover, this study proposes an efficient methodology for obtaining practical data and seeks to dissect the mechanism behind phase transformation by predicting transformation curves and microstructures.

## 2. Methodology

### 2.1. α Ferrite Transformation and Carbon Concentration

**Figure 1:**
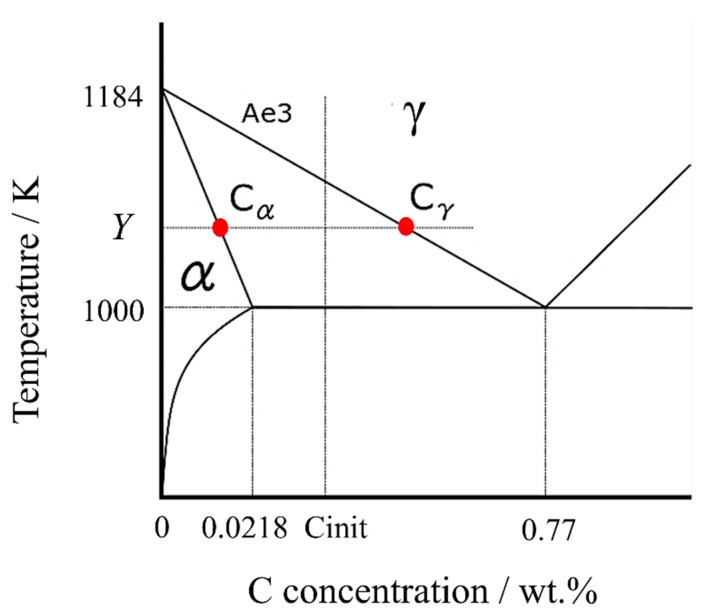
Fe-C binary phase diagram in the vicinity of ferrite transformation.

According to the Fe-C binary phase diagram in Figure 1, assuming that the Ae3 line and the α solid phase line, where the α transformation begins, are straight lines, the lines can be expressed by the following equations, respectively.

(1) $$Ae3\;line:y=\frac{1184-1000}{0.0077-0}x+1184$$

(2) $$\alpha \;solid\;phase\;line:y=\frac{1184-1000}{0.000218-0}x+1184$$

The carbon concentration in the γ phase, $C_{\gamma }$, can be calculated using the carbon concentration in the α phase [41], $C_{\alpha }$,

(3) $$C_{\gamma }=\frac{0.0077}{0.000218}C_{\alpha }$$

At any temperature *Y* (lower than the Ae3 point), the carbon concentrations in α and γ phases can be expressed as

(4) $$C_{\alpha }=\frac{0.000218}{1184-1000}\left(Y-1184\right)$$

(5) $$C_{\gamma }=\frac{0.0077}{1184-1000}\left(Y-1184\right)$$

### 2.2. Nucleation

**Figure 2:**
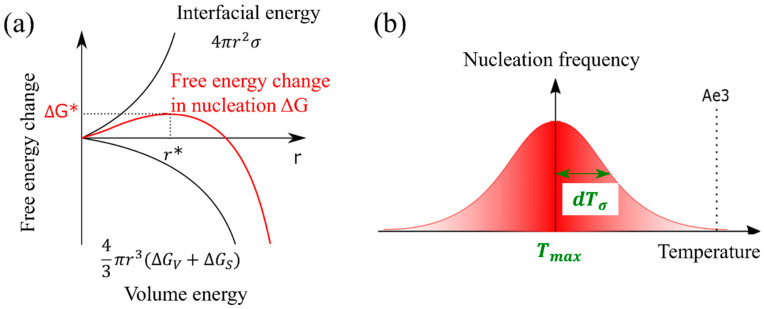
(**a**) Nucleation-free energy changes as a function of the interfacial energy and volume energy per volume, (**b**) temperature-dependent nucleation frequency settings and parameters.

The nucleation of the α phase in the γ phase induces the change in the nucleation free energy, denoted as ∆G. This change can be mathematically represented by Equation (8), which is a composite expression involving both the interfacial energy (as described in Equation (6)) and the volume energy equations (as expressed in Equation (7)) [42]. The nucleation frequency can be expressed in Equation (9). Figure 2a shows the nucleation-free energy changes as a function of the interfacial energy and volume energy per volume.

(6) $$\mathrm{Interfacial}\;\mathrm{energy}=4\pi r^{2}\sigma$$

(7) $$\mathrm{Volume}\;\mathrm{energy}=-\frac{4}{3}\pi r^{3}\left(∆G_{v}+∆G_{s}\right)$$

(8) $$∆G=-\frac{4}{3}\pi r^{3}\left(∆G_{v}+∆G_{s}\right)+4\pi r^{2}\sigma$$

(9) $$\mathrm{Nucleation}\;\mathrm{frequency}\propto exp\left(-\frac{∆G+∆G_{d}}{kT}\right)$$

where r is grain diameter, σ is interfacial energy per volume, $∆G_{v}$ is the free energy change per volume, $∆G_{s}$ is the elastic strain energy change per volume, $∆G_{d}$ is the activation energy of diffusion.

The α nucleation frequency, as described in Equation (10), exhibits a Gaussian distribution pattern that is temperature dependent. In this equation, $T_{max}$ represents the temperature at which the probability of nucleation is maximized, and $dT_{\sigma }$ denotes the standard deviation [35]. Furthermore, the assumption that the α nucleation frequency follows a Gaussian function is justified by the resemblance of the change in nucleation-free energy, as depicted by the solid red line in Figure 2b, to a Gaussian function.

(10) $$\mathrm{Nucleation}\;\mathrm{frequency}=\frac{1}{\sqrt{2\pi \times dT_{\sigma }}}exp\left(-\frac{{\left(T-T_{max}\right)}^{2}}{2\times {\left(dT_{\sigma }\right)}^{2}}\right)$$

Furthermore, it is essential to consider a time delay before nucleation attains a stable state, a phenomenon commonly referred to as the incubation period of nucleation [5,43]. In this study, this incubation period was incorporated into the analysis using Equations (11) and (12). In Equation (10), the nucleation frequency was replaced by $J^{*}$, representing the steady-state nucleation frequency.

(11) $$J^{*}=J_{s}^{*}exp\left(-\frac{\tau }{t}\right)$$

(12) $$\tau =\frac{8kT\sigma a^{4}}{v_{a}^{2}{\left(∆G_{v}\right)}^{2}Dx_{B}}$$

where k is Boltzmann’s constant, T is temperature, σ is interfacial energy per unit volume, a is the lattice constant, $v_{a}$ is the molar fraction of a phase, $∆G_{v}$ is the driving force for nucleation, D is the diffusion constant, $x_{B}$ is the solute atom mole fraction.

The driving force for nucleation is approximated as the degree of undercooling. In addition, some coefficients of τ are assumed to be constant in this study as the incubation period is constant, as shown in Equation (13).

(13) $$\tau =(incubation\;period\;constant)\frac{T}{{\left(∆G_{v}\right)}^{2}Dx_{B}}$$

### 2.3. Diffusion

In the initial γ structure, the carbon concentration was assumed to be uniformly distributed in each cell. As depicted in the phase diagram of carbon steel in Figure 1, the α phase has a lower concentration of solid solute carbon than the γ phase. Consequently, when α nucleation takes place, the excess carbon that cannot be dissolved into the solid solution is expelled, increasing the local carbon concentration in the vicinity. This alteration in carbon concentration sets up a concentration gradient, driving carbon diffusion.

To address this diffusion process, an explicit finite difference method was employed based on Fick’s 2nd law of diffusion. The equations representing Fick’s second law (Equation (14)) and the explicit method (Equation (15)) in two dimensions are as follows. In these equations, $C_{i,j}$ represents the carbon concentration in the cell at coordinates (*i*, *j*), D stands for the diffusion coefficient, and t represents time. The calculation of the carbon diffusion coefficient was carried out using equation (Equation (16)).

(14) $$\frac{\partial c}{\partial t}=D\frac{\partial_{c}^{2}}{\partial_{x^{2}}}+D\frac{\partial_{c}^{2}}{\partial_{y^{2}}}$$

(15) $$C_{C_{i,j}}^{t+∆t}=C_{C_{i,j}}^{t}+D\times ∆t\left(\frac{C_{C_{i-1,j}}^{t}-2C_{C_{i,j}}^{t}+C_{C_{i+1,j}}^{t}}{∆x^{2}}-\frac{C_{C_{i-1,j}}^{t}-2C_{C_{i,j}}^{t}+C_{C_{i+1,j}}^{t}}{∆y^{2}}\right)$$

(16) $$D=6.68\times 10^{-5}exp\left(-\frac{157000}{8.314\times T}\right)$$

In addition to the explicit method, an implicit method is also available for solving the difference method. However, the implicit method involves solving complex simultaneous linear equations, leading to opting for the explicit method. It is worth noting that the solution obtained through the explicit method is subject to a stability condition, as depicted in Equation (17) [44].

(17) $$0\leq D\frac{∆t}{∆x^{2}}+D\frac{∆t}{∆y^{2}}\leq \frac{1}{2}$$

Utilizing diffusion calculations as transition rules within the CA method, simultaneous calculations were conducted for the entire microstructure and predicted the state at the subsequent calculation step. The von Neumann neighborhood approach was employed to address the diffusion equation. Notably, within the γ grains, there is an increase in carbon concentration due to α nucleation. Therefore, following the initiation of the α phase transformation, a carbon concentration gradient occurs within the γ grains. This gradient was established based on the assumption that the carbon concentration in the interface cell would decrease by a specific factor compared to the adjoining cell.

### 2.4. Grain Growth

In the CA mode, the grain growth was calculated using the following Equation (18), where $C_{avg}$ represents the average carbon concentration in the interfacial cell, $∆f_{s}$ indicates the α transformation rate of the interfacial cell per calculation step. When the sum of the α transformation rates in the interfacial cells surpasses 1, signifying the completion of α transformation, the interfacial cell is designated to move [45,46,47,48]. For cells in contact with multiple types of α-grains simultaneously, the choice of α-grain orientation was determined probabilistically by employing a random number.

(18) $$∆f_{s}=\frac{C_{\gamma }-C_{avg}}{C_{\gamma }-C_{\alpha }}$$

### 2.5. Martensitic Transformation

The γ phase contains a significantly higher carbon concentration in solid solution than the α phase. So, when γ experiences rapid cooling, it undergoes shear deformation without the diffusion of carbon atoms, resulting in martensite formation. This characteristic is called diffusionless transformation or shear-type transformation. In this modeling, when the α volume fraction obtained from the lever principle of the phase diagram was exceeded, quenching was assumed, and retained austenite was assumed to have transformed into martensite.

### 2.6. Parameter Estimation by Bayesian Optimization

To clarify the relationship between inputs and outputs, it is necessary to optimize the functions for well linking them.

An illustrative approach for obtaining output values involves considering all possible input values, commonly known as grid search. In this method, results are determined through exhaustive computation, which significantly escalates the computational cost. Consequently, an alternative method called random search, which computes input values randomly, has been proposed. However, due to its stochastic nature, no mathematical proof exists substantiating a reduction in the number of trials [49].

To address this challenge and pursue more efficient calculations, Bayesian optimization emerges as a valuable solution. Particularly in materials engineering, it is recognized as a data-driven approach that seamlessly integrates data collection and analysis [50]. Bayesian optimization is characterized by its capacity to optimize black-box functions efficiently by orchestrating a search and evaluation process based on the information acquired from training data. In this study, we aimed to enhance the accuracy of our model by seeking optimal parameters through the prediction of an evaluation function.

An evaluation function was defined, where a smaller value indicates a higher level of accuracy in the developed model. It can be expressed as Equation (19) with the input value denoted as $x^{*}$. Gaussian optimization leverages regression to optimize hyperparameters while reducing the number of trial runs. About the Gaussian process, the joint distribution of x ($x_{1}$, $x_{2}$, $x_{3}$,…, $x_{N}$) in $f\left(x\right)$ follows a Gaussian distribution [51]. It is important to note that the joint distribution of variables A and B signifies the probability of each variable taking on specific values. Also, the sum of possibilities related to only a particular random variable is referred to as the marginal distribution. Here, if the objective variable to be observed includes independent noise $\varepsilon_{n}$ at each observation point, it can be expressed as Equation (20).

(19) $$x^{*}=\alpha rgminf\left(x\right)$$

(20) $$t_{n}=y_{n}+\varepsilon_{n}$$

When $\varepsilon_{n}$ conforms to a Gaussian distribution, the accuracy parameter β will be employed.

(21) $$p\left(t_{n}|y_{n}\right)=N\left(t_{n}|y_{n},\beta^{-1}\right)$$

Here, if a joint distribution was expressed using $I_{n}$ representing the N × N unit matrix, then

(22) $$p\left(t|y\right)=N\left(t|y,\beta^{-1}I_{n}\right)$$

The marginal distribution is calculated using the kernel function *K*,

(23) $$p\left(y\right)=N\left(y|0,K\right)$$

The marginal distribution of ***t***, a Gaussian distribution, can be expressed as Equation (24).

(24) $$p\left(t\right)=\int p\left(t|y\right)p\left(y\right)dy=N\left(t|0,C\right)$$

(25) $$C_{nm}=k\left(x_{n},x_{m}\right)+\beta^{-1}\delta_{nm}$$

According to the training data input x ($x_{1}$, $x_{2}$, $x_{3}$,…, $x_{N}$), the predicted distribution $p\left(t_{N+1}|t_{N}\right)$ of the new input data $x_{N+1}$ can be obtained from Equation (24). By dividing the predicted distribution covariance matrix $C_{N+1}$, let $k_{n}=k\left(x_{N},x_{N+1}\right)$, $c=k\left(x_{N+1},x_{N+1}\right)+\beta^{-1}$, then,

(26) $$p\left(t_{N+1}\right)=N\left(t_{N+1}|0,C_{N+1}\right)$$

(27) $$C_{N+1}=\left(\begin{array}{cc} C_{n} & k\\ k & c \end{array}\right)$$

(28) $$\mathrm{Average}:\;\mu \left(x_{N+1}\right)=k^{T}C_{N}^{-1}t$$

(29) $$\mathrm{Dispersion}:\;\sigma^{2}\left(x_{N+1}\right)=c-k^{T}C_{N}^{-1}k$$

Equations (28) and (29) can predict the noise ε in Equation (20) for Gaussian process regression.

The Bayesian optimization method estimates input values that satisfy the conditions described in Equation (19) by utilizing the regression curve derived from Gaussian process regression. Initially, it predicts the response variable based on the available training data and previous results. Subsequently, using an indicator known as the acquisition function assesses the likelihood of obtaining a value that surpasses the previously optimal result. The method then proceeds to search for input values that fulfill these conditions. Increasing the number of trials leads to the accumulation of additional training data, which, in turn, narrows down the range of predicted functions, ultimately resulting in more reliable predictions. It is worth noting that certain aspects, particularly Gaussian process regression, have been omitted from this study as they are not the primary focus of our research. Comprehensive resources can be found in widely recognized textbooks. Various acquisition functions have also been proposed in the literature [52,53,54,55].

**Figure 3:**
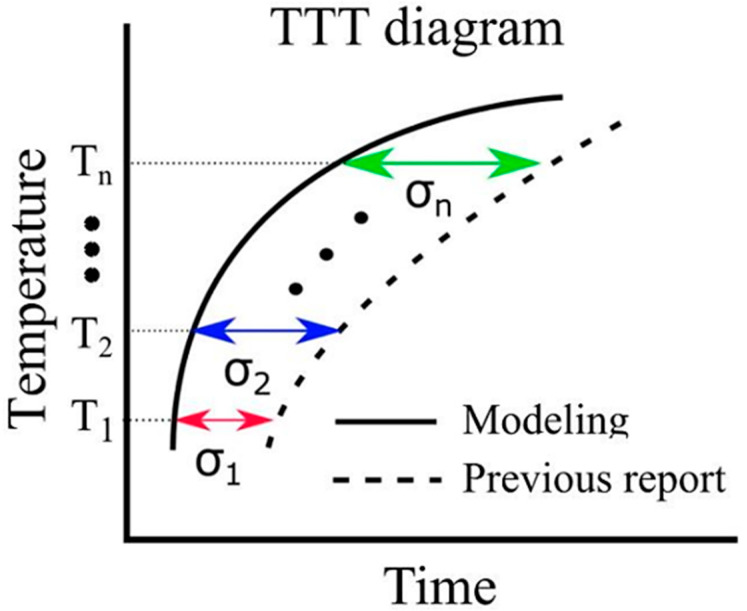
Calculation of evaluation function in the TTT diagram.

Thermal transformation diagrams, known as time–temperature–transformation (TTT) diagrams and continuous cooling transformation (CCT) diagrams, serve as crucial tools for comprehending phase transformation properties [56]. The ability to predict these TTT and CCT diagrams holds the potential for substantial advancements in steel development. While Enomoto et al. successfully made such predictions, underscoring the need for further insights into the α-phase transformation, which remains a significant research challenge [57]. In this model, the function $f\left(x\right)$ in Equation (19) represents the error between the simulation results and the literature values pertaining to the TTT diagram. The evaluation function serves the purpose of assessing this error. In Figure 3, it is important to note that $\sigma_{n}$ and $x_{i}$ correspond to the value on the horizontal axis of the TTT diagram at time i. Due to the unique attributes of each evaluation function, the root mean squared logarithmic error (RMSLE) was employed. The evaluation function was computed based on results obtained at four different temperatures: 900, 950, 1000, and 1050 K. Additionally, the RMSLE formula in Equation (30) utilizes $log(1+x_{i})$ to prevent situations where the evaluation function becomes incalculable due to log(0) being undefined. ${\hat{x}}_{i}$ indicates the reference value at time i. However, it is crucial to mention that in our model, the phase transformation does not solely commence at the initial nucleation corresponding to 0 s. Thus, the logarithm function remains within its defined range. Therefore, as an evaluation function that is more intuitive than RMSLE, we introduced the $RMSE_{v}$ evaluation function expressed in Equation (31). We obtained results using both RMSLE and $RMSE_{v}$ and it is evaluated which one is more suitable. As indicated by the equations, it can be concluded that the closer the values of RMLSE and RMSEV are to 0, the more closely the literature values align with the model results.

(30) $$RMSLE=\sqrt{\frac{1}{n}\sum_{i=1}^{n}{\left(log\left(1+x_{i}\right)-\left(\log(1+{\hat{x}}_{i}\right)\right)}^{2}}$$

(31) $$RMSE_{v}=\sqrt{\frac{1}{n}\sum_{i=1}^{n}{\left(\log\left(x_{i}\right)-\log\left({\hat{x}}_{i}\right)\right)}^{2}}$$

## 3. Results and Discussion

### 3.1. Effect of Individual Parameter

The primary parameters in our study include the function that links the computational step to real-time and specific parameters associated with nucleation. The former parameterizes converting computational steps into real-time units by multiplying them by a coefficient. This coefficient corresponds to ∆t in Equation (17). According to this equation, it is imperative to minimize this value to meet the stability condition. However, if it becomes significantly smaller than the critical threshold, it results in longer calculation times. Such an exceedingly small value may diminish the advantages of the CA method, rendering it less efficient. Therefore, we initially set this coefficient to 0.1 and only adjusted it when the stability conditions were not achieved.

**Figure 4:**
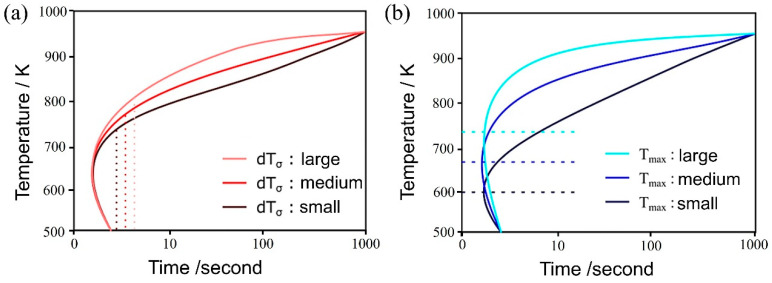
Influence of (**a**) $dT_{\sigma }$ and (**b**) $T_{max}$ on the TTT diagram.

Figure 4a,b illustrate the influence of both $dT_{\sigma }$ and $T_{max}$ on the TTT diagram, respectively. As the parameter $dT_{\sigma }$ decreases, noticeable changes occurred in the curvature around the nose of the TTT diagram, both at the initiation and completion of the transformation, resulting in a more distinct nose feature. This phenomenon arises from the reduction in standard deviation, which, in turn, leads to an increased difference between the temperature at which the nucleation frequency reaches its maximum and the holding temperature. This differential temperature alteration significantly impacts the nucleation frequency.

Conversely, the parameter $T_{max}$ has a notable influence on the TTT diagram. As its value decreases, the nose position in the TTT diagram fluctuates at both the onset and conclusion of the transformation. This occurs because the initiation of the transformation involves carbon diffusion, and the diffusion rate is temperature-dependent, as evidenced by Equation (14). Therefore, the extent of change in $T_{max}$ does not necessarily correspond directly to the change in the nose position [58].

Regarding the incubation period constant, an increase in its value is expected to shift the TTT diagram to the right, resulting in a slower initiation and completion of the transformation. This is attributed to the fact that following Equation (7), the α nucleation frequency gradually increases in a time-dependent manner until it reaches a steady state. However, the simulation results exhibit a pattern similar to that observed with $dT_{\sigma }$, where an increase in the incubation period constant causes a decrease in the TTT diagram. Consequently, the curvature around the nose becomes more pronounced, rendering the nose feature more distinct.

### 3.2. Optimal Value of Each Parameter Obtained by Bayesian Optimization

One distinctive feature of Bayesian optimization is its tendency for search parameters to converge towards a constant value. However, in this model, since the nucleation frequency is influenced by stochastic factors, there is no direct one-to-one correspondence between each parameter and its resulting TTT diagram or organizational chart. Consequently, the frequency of parameter searches is regarded as a critical aspect of Bayesian optimization within this study. As a result, in our research, determining the optimal value for each parameter is not solely based on identifying the minimum RMSLE (root mean squared logarithmic error). Instead, we adopt a holistic approach considering the parameter search frequency within the Bayesian loop, the obtained TTT diagram, and the RMSLE value. Our judgment is based on these three perspectives. Furthermore, guided by this approach, we establish the optimal value as a range rather than a singular point, recognizing the inherent variability and complexity of the system under consideration.

**Figure 5:**
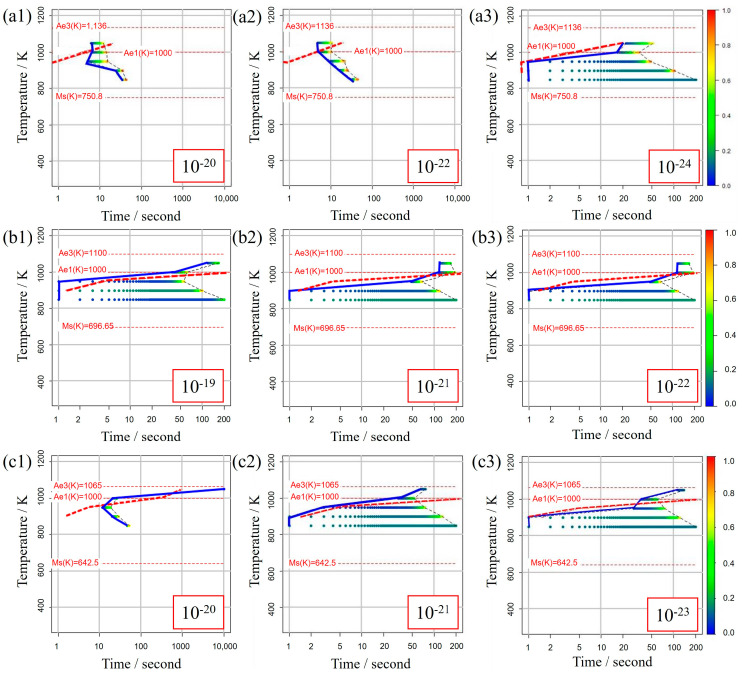
TTT diagrams obtained from different values of the incubation period constant in (**a1**–**a3**) Fe−0.20 wt.%C, (**b1**–**b3**) Fe−0.35 wt.%C, (**c1**–**c3**) Fe−0.50 wt.%C. The incubation period constants are shown in the inserted red frames at the lower right in each diagram.

Figure 5 illustrates the TTT diagrams obtained from different values of the incubation period constant across three distinct carbon concentration levels. The color bars represent the α volume fraction versus time at various holding temperatures as simulated in the model. Furthermore, the curve denoting the initiation of α-phase transformation is depicted by a solid blue line for our study’s results and a dashed red line for previous experimental results. This color scheme is consistently applied in subsequent TTT diagrams. From these results, it becomes evident that an incubation period constant of $10^{-24}$ is well-suited for Fe-0.20 wt.%C, as shown in Figure 5a1–a3. Similarly, moving on to Fe-0.35 wt.%C, as shown in Figure 5b1–b3, the RMSLE value is minimized, and the overall shape of the TTT diagram aligns appropriately with an incubation period constant of $10^{-19}$. However, for Fe-0.50 wt.%C, as shown in Figure 5c1–c3, even though the RMSLE value reaches its minimum at an incubation period constant of $10^{-20}$, the temperature at the nose position, a critical feature in the TTT diagram, significantly deviates from the literature value. In light of this, we conclude that an incubation period constant yielding a lower RMSLE value of $10^{-21}$ is more suitable in this case.

**Figure 6:**
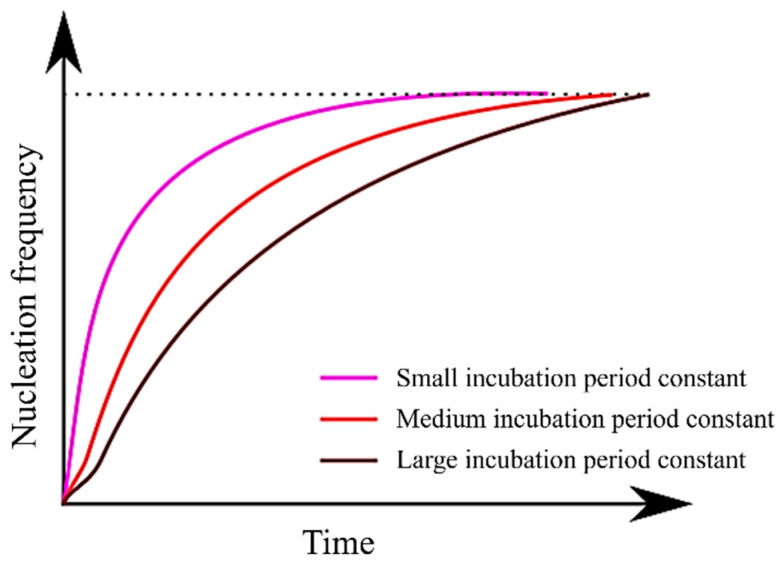
Relationship between incubation period constant and nucleation frequency.

Figure 6 illustrates the relationship between the incubation period constant and the nucleation frequency, as described in Equation (13). It is evident from Equation (13) that as the incubation period constant increases, the value of τ also increases. Consequently, it can be inferred that an increase in τ prolongs the time required to attain a steady-state nucleation frequency. Moreover, since Equation (13) incorporates a temperature term (T) in its numerator, it becomes apparent that τ assumes larger values at higher temperatures. In essence, an increase in the incubation period constant is expected to have an effect akin to a decrease in $dT_{\sigma }$. This explains why the TTT diagram does not simply shift to the right.

**Figure 7:**
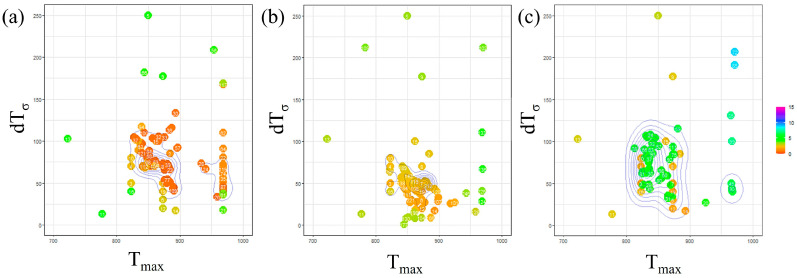
Exploration of optimal parameter values using Bayesian loops in (**a**) Fe-0.20 wt.%C, (**b**) Fe-0.35 wt.%C, (**c**) Fe-0.50 wt.%C.

Figure 7 presents the outcomes of Bayesian optimization involving two parameters, $T_{max}$ and $dT_{\sigma }$, with the incubation period constant determined for each carbon concentration. In this figure, the parameters investigated in each Bayesian loop are represented on a two-dimensional plot with $T_{max}$ on the horizontal axis and $dT_{\sigma }$ on the vertical axis. The RMSLE value corresponding to each parameter combination is depicted using a color bar. Additionally, contour lines are included to indicate the density of the parameter search. Based on this representation, we identify the parameter values associated with a lower RMSLE and those exhibiting clustered contour lines as the optimal choice.

Concerning the parameter $T_{max}$, from a macroscopic perspective, it is apparent that the values tend to cluster around 850 K across all carbon concentrations. This proximity to the nose position in the TTT diagram suggests that we were able to identify a reasonable value, as discussed, regarding the influence of $T_{max}$. However, when observed microscopic, the values tend to be relatively high for Fe-0.35 wt.%C and relatively low for Fe-0.50 wt.%C. The specific behavior of Fe-0.35 wt.%C is believed to be interconnected with the values of other parameters, and we will delve into this further in subsequent discussions, along with an analysis of the $dT_{\sigma }$ and incubation period constant values. Regarding Fe-0.50 wt.%C, the slower onset of α-phase transformation in the temperature range of 950 K or higher compared to other carbon concentrations led us to attempt to replicate this behavior by lowering the nose of the TTT diagram. This adjustment is conceivable as a potential explanation for the observed behavior.

Regarding the parameter $dT_{\sigma }$, when taking a macroscopic view, the results indicate no substantial variation in the value across all carbon concentrations. Nevertheless, at a microscopic level, it was lower for Fe-0.35 wt.%C. This observation is likely a consequence of our efforts to sustain a specific level of nucleation frequency due to the high value sought for $T_{max}$.

Furthermore, upon examining the overall frequency of parameters appearing in the Bayesian loop, it was noted that the parameters exhibited relatively more dispersion for Fe-0.50 wt.%C. This occurrence is likely attributed to the fact that the evaluation function yields relatively higher values than other carbon concentrations. Consequently, this simulation is highly accurate for carbon concentration conditions, where α-phase transformation occurs swiftly across all temperature ranges.

Table 1 summarizes the optimal values for the three parameters mentioned above and contrasts them with a model that does not consider the incubation period of nucleation. The most notable distinction between the two models is that in the model accounting for the incubation period, a significant correlation exists between carbon concentration and the constant incubation period. Conversely, in the model that omits the incubation period, $dT_{\sigma }$ exhibits a strong relationship.

The results indicate a significant dependency of the constant incubation period on carbon concentration variations. Specifically, the lowest carbon concentration, Fe-0.20 wt.%C, exhibits the smallest value compared to other carbon concentrations. This aligns with expectations since the α transformation initiates earlier in this composition. Conversely, the value for Fe-0.35 wt.%C is more significant than Fe-0.50 wt.%C. This might seem inconsistent with the previously mentioned observation that an increase in the nucleation latency constant corresponds to behavior akin to a decrease in $dT_{\sigma }$. However, it is noteworthy that the optimal $T_{max}$ values for both carbon concentrations in Table 1 are smaller for Fe-0.50 wt.%C than for Fe-0.35 wt.%C. Consequently, it can be inferred that the nucleation frequency, as given by Equation (10), is relatively lower for Fe-0.50 wt.%C. This observation likely contributes to the more significant incubation period constant for Fe-0.35 wt.%C.

**Figure 8:**
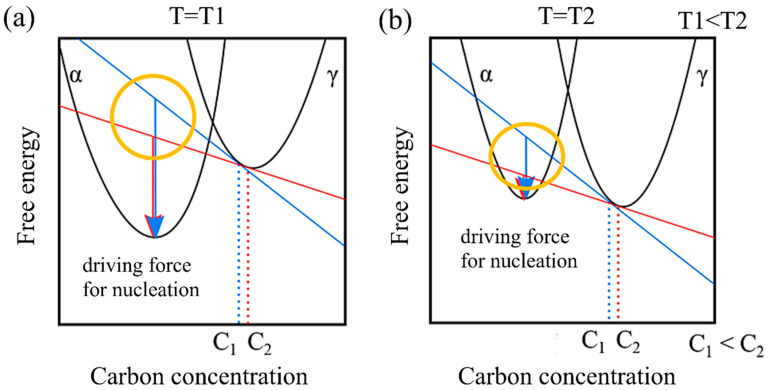
Carbon concentration and driving force for nucleation based on the viewpoint of free energy at (**a**) low temperature and (**b**) high temperature.

Additionally, in a model that does not account for the incubation period, $T_{max}$ remains unaffected by carbon concentration, while $dT_{\sigma }$ tends to decrease as carbon concentration increases. This behavior can be attributed to the driving force of nucleation. Figure 8 illustrates the relationship between the driving force for nucleation and carbon concentration from the perspective of free energy. Let us consider the free energies of the α and γ phases at a specific temperature T_1_ and a higher temperature T_2_. At T_1_, in contrast to T_2_, the free energy of the α phase is relatively lower than that of the γ phase. Moreover, assuming specific carbon concentrations C_1_ and C_2_, the respective driving forces for nucleation are represented by the arrows. Comparing these at each temperature reveals that the driving force for nucleation decreases as the temperature increases. Furthermore, when comparing the differences in the driving force for nucleation resulting from different carbon concentrations (the area circled in yellow), it becomes evident that the impact of the relative difference in carbon concentration on the driving force for nucleation becomes more pronounced at higher temperatures.

**Figure 9:**
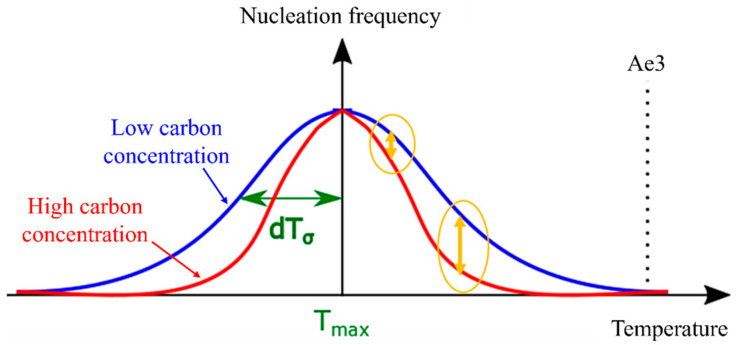
Relationship between carbon concentration and dTσ.

As temperature increases, it is evident that the driving force for nucleation becomes more reliant on carbon concentration. This relationship is translated into Figure 9 when applied to Equation (10). With $dT_{\sigma }$ decreasing as carbon concentration increases, the nucleation frequency exhibits minimal dependence on carbon concentration in the low-temperature range. However, the model can accurately reproduce changes that demonstrate a strong dependency on carbon concentration in the high-temperature range. Consequently, in models that do not account for the nucleation incubation period, the value of $dT_{\sigma }$ tends to decrease as carbon concentration increases.

### 3.3. TTT Diagram

**Figure 10:**
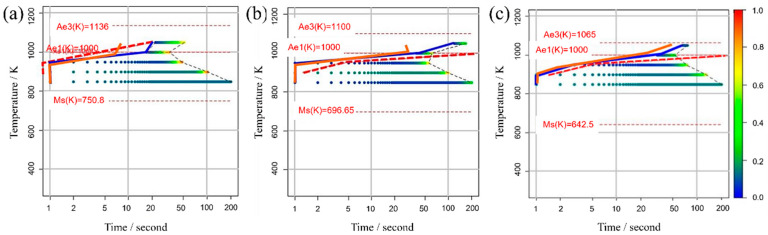
Comparison of the TTT diagrams using optimal parameters with and without consideration of nucleation incubation period in (**a**) Fe-0.20 wt.%C, (**b**) Fe-0.35 wt.%C, (**c**) Fe-0.50 wt.%C. The blue solid line represents the simulation result when considering the nucleation incubation period. The orange solid line represents the result without considering the nucleation incubation period. The red dashed line represents the previous results [45].

It is important to note that among the optimal values for each parameter, the one with the minimum RMSLE was utilized in this illustration. Additionally, a comparison is presented between the obtained TTT diagram and the results from a model that disregards the nucleation incubation period. The solid orange line represents the outcome of the TTT diagram obtained via Bayesian optimization in a model that does not consider the nucleation incubation period, as shown in Figure 10.

Regarding the outcomes achieved with the model that incorporates the incubation period, it can be affirmed that we were successful in obtaining TTT diagrams that closely align with previous experimental results for each carbon concentration. Notably, in the temperature range below 950 K, the error was minimal, with deviations of only a few seconds, resulting in high predictive accuracy. Although there was no significant difference observed for Fe-0.20 wt.%C, Fe-0.35 wt.%C, and Fe-0.50 wt.%C, in the temperature range above 950 K, notable improvements in accuracy were evident when compared to the results obtained without considering the incubation period. This underscores the significance of accounting for the nucleation incubation period in this model.

The primary reason for this improvement is the stochastic selection of nucleation sites in this model. In models that do not consider the nucleation incubation period, α nucleation is determined solely by probability theory. Consequently, when the nucleation frequency is low, simulations conducted over several hundred seconds (or several thousand calculation steps) can yield scenarios where nucleation commences early by chance or, conversely, where nucleation fails to initiate. Indeed, based on the literature values [59], it has been observed that, depending on carbon concentration, it takes approximately 100 s for transformation to commence at 1050 K after isothermal holding. This effect is considered to be particularly significant. Moreover, in high-temperature regions, elemental diffusion occurs more rapidly compared to low-temperature areas, making it more likely for phase transformation to begin once nucleation initiates. Therefore, models that do not account for the nucleation incubation period are deemed less capable of reliably predicting transformation in high-temperature regions. Conversely, in the model incorporating the nucleation incubation period, rendering the nucleation frequency contingent on the holding time becomes possible. This capability is believed to mitigate the influence of probabilistic factors on nucleation, allowing for more precise control of phase transformation.

The model considering the nucleation incubation period reveals a dependence between carbon concentration and the incubation period constant, whereas in the model neglecting the nucleation incubation period, $dT_{\sigma }$ is linked to carbon concentration. Consequently, it is inferred that the model incorporating the nucleation incubation period has enhanced the predictive accuracy of the TTT diagram.

### 3.4. Evaluation Function and TTT Diagram

**Figure 11:**
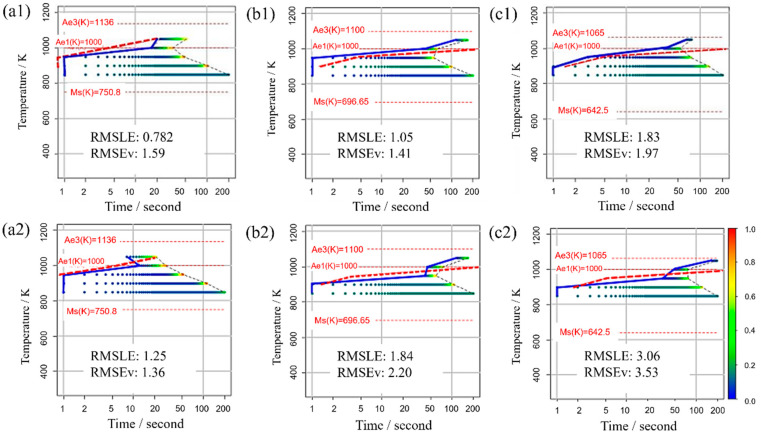
TTT diagram obtained using RMSLE and RMSEV as the evaluation functions in Bayesian optimization in (**a1**,**a2**) Fe-0.20 wt.%C, (**b1**,**b2**) Fe-0.35 wt.%C, (**c1**,**c2**) Fe-0.50 wt.%C. The blue solid line represents the simulation result. The red dashed line represents the previous results [44].

Figure 11 displays the TTT diagram obtained using RMSLE and RMSEV as the evaluation functions in Bayesian optimization, along with the respective values of each evaluation function. For this scenario, appropriate values were utilized for all incubation period constants. The upper portion presents the Bayesian optimization evaluation function using RMSLE, while the lower part showcases the Bayesian optimization evaluation function using RMSEV. Additionally, both RMSLE and RMSEV values were computed from each TTT diagram. The results reveal that when RMSLE is employed as the evaluation function, it yields smaller values for both evaluation functions across all carbon concentrations. Consequently, RMSLE is the more suitable evaluation function for Bayesian optimization in this model.

**Figure 12:**
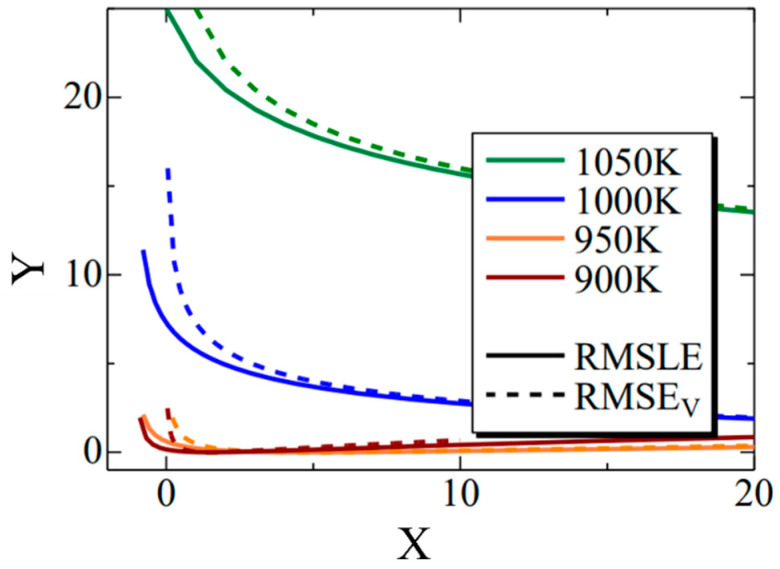
The Σ interior function in the RMSLE and RMSE_V_ equations. The black solid and dashed lines indicate the RMSLE and RMSLE_V_, respectively. The green, blue, orange, and brown lines indicate the temperature of 1050 K, 1000 K, 950 K and 900 K, respectively.

Figure 12 shows the Σ interior function in the RMSLE and RMSEV equations. Additionally, the term representing the literature value was substituted with the value of Fe-0.50 wt.%C. The horizontal axis (x) corresponds to time (*t*) in Equation (11), while the vertical axis (y) corresponds to the nucleation frequency. The solid line represents RMSLE, and the dashed line represents RMSEV. Although not explicitly shown in this figure for clarity, RMSLE approaches x = −1, while RMSEV approaches x = 0. Notably, in regions where the value of x is relatively tiny, RMSEV exhibits larger values than RMSLE. Furthermore, this trend becomes more pronounced at higher temperatures. Consequently, it can be inferred that RMSEV tends to overestimate the vicinity of x = 0, particularly at elevated temperatures. This effect likely contributed to the search for parameters that delay the onset of α-phase transformation in the high-temperature region. In summary, using RMSLE as an evaluation function is deemed to have a similar effect as considering the incubation period of nucleation.

### 3.5. Alpha Phase Transformation Structure

**Figure 13:**
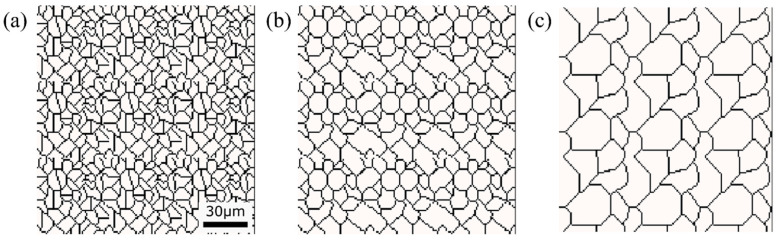
Initial microstructure virtuality with different average γ grain sizes: (**a**) 5.01 μm, (**b**) 8.13 μm, (**c**) 15.00 μm.

**Figure 14:**
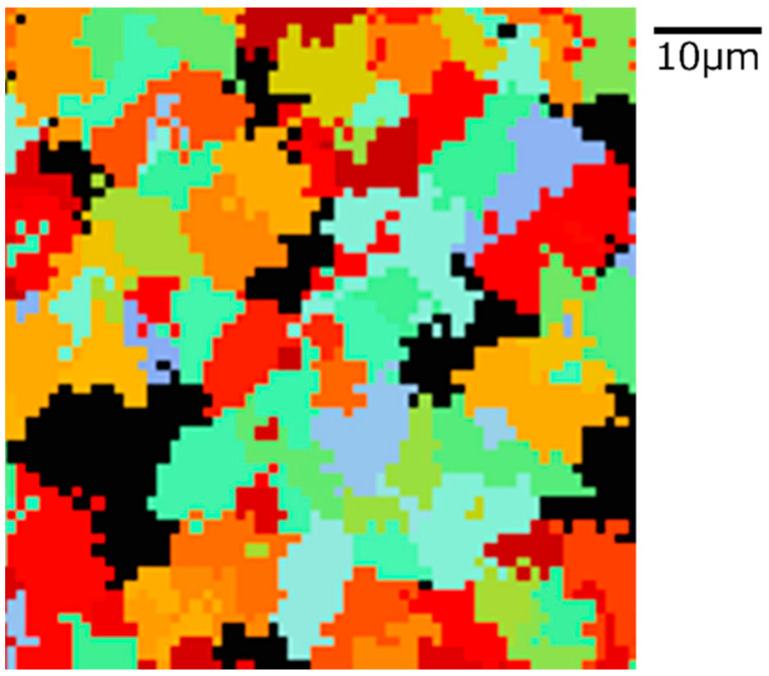
Microstructure simulation result of Fe-0.20 wt.%C after annealing at 950 K.

The state of α phase transformation is monitored at each calculation step using the optimal parameter values specific to each carbon concentration. The virtual initial γ structures employed in these calculations encompass three distinct types with varying initial γ grain sizes, as illustrated in Figure 13. It is important to note that the black regions represent the boundaries of the γ grains. Figure 14 displays the microstructural outcomes of Fe-0.20 wt.%C after holding at 950 K, maintaining the same microstructure as a previous experiment result [60]. The crystal orientation of α grains is depicted using color bars, where colored regions signify α grains, while white regions represent γ grains. It is worth mentioning that in the earlier research, black grains corresponded to γ, and white grains represented α. These results show that realistic shapes and scales of α grains have been accurately predicted for all carbon concentrations, demonstrating favorable alignment with previous studies.

**Figure 15:**
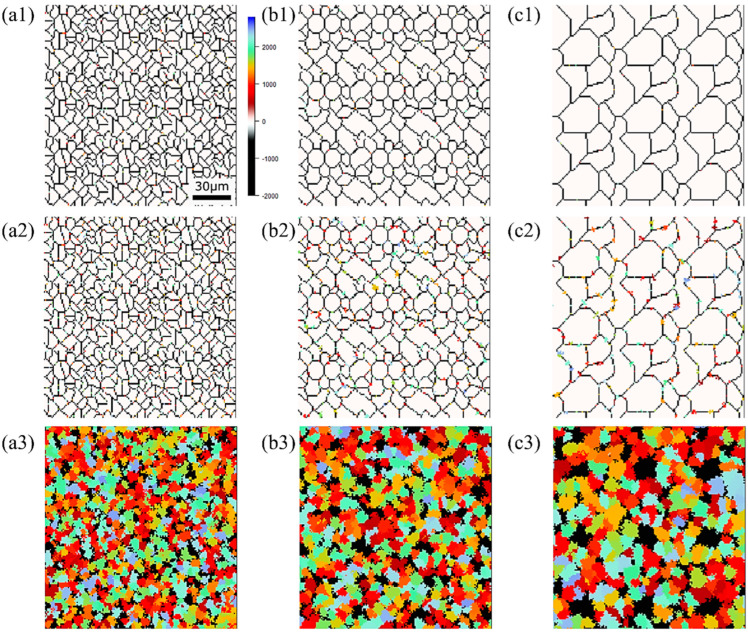
Microstructure during α transformation in Fe−0.20 wt.%C with different initial average γ grain sizes (**a**) 5.01 μm, (**b**) 8.13 μm, (**c**) 15.00 μm after annealing at 1000 K (**a1**,**b1**,**c1**) at the beginning of annealing process, (**a2**,**b2**,**c2**) at the beginning of phase transformation with the volume fraction of α phase 3%, (**a3**,**b3**,**c3**) after completion of phase transformation.

**Figure 16:**
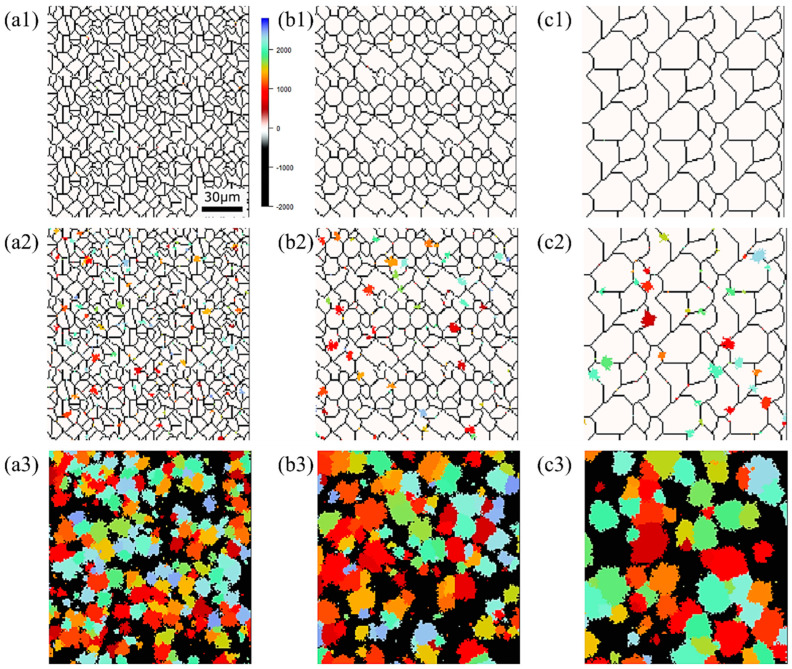
Microstructure during α transformation in Fe−0.35 wt.%C with initial different average γ grain sizes (**a**) 5.01 μm, (**b**) 8.13 μm, (**c**) 15.00 μm after annealing at 1000 K (**a1**,**b1**,**c1**) at the beginning of annealing process, (**a2**,**b2**,**c2**) at the beginning of phase transformation with the volume fraction of α phase 3%, (**a3**,**b3**,**c3**) after completion of phase transformation.

**Figure 17:**
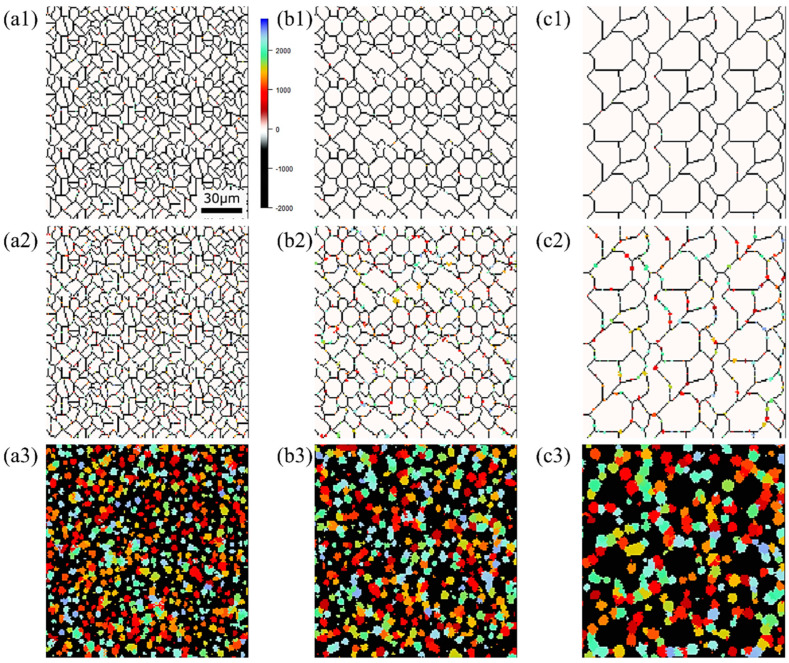
Microstructure during α transformation in Fe−0.50 wt.%C with initial different average γ grain sizes (**a**) 5.01 μm, (**b**) 8.13 μm, (**c**) 15.00 μm after annealing at 1000 K (**a1**,**b1**,**c1**) at the beginning of annealing process, (**a2**,**b2**,**c2**) at the beginning of phase transformation with the volume fraction of αphase 3%, (**a3**,**b3**,**c3**) after completion of phase transformation.

**Figure 18:**
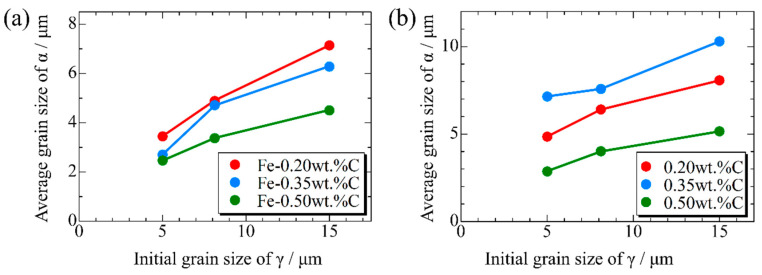
Relationship between initial γ grain size and average grain size of α after phase transformation in the samples with different carbon concentrations after annealing at (**a**) 950 K and (**b**) 1000 K.

Figure 15, Figure 16 and Figure 17, respectively, show the microstructure during α transformation in Fe-0.20 wt.%C, Fe-0.35 wt.%C, and Fe-0.50 wt.%C with different average γ grain sizes as mentioned in Figure 13 after annealing at 1000 K. It also displays the stages of the transformation process, such as at the beginning of annealing and transformation and the completion of phase transformation. These results were consistent with those obtained at 900, 950, and 1050 K, where the TTT diagram was predicted using this model. Additionally, Figure 18a,b illustrate the relationship between the initial γ grain size and the average α grain size upon completion of the α transformation at 950 and 1000 K. It is evident from these findings that the α average grain size diminishes as the initial γ grain size decreases. Moreover, when investigating the connection between carbon concentration and the α average grain size upon completing the α phase transformation, it was observed that at a holding temperature of 950 K, the α average grain size decreases with increasing carbon concentration, aligning with conventional findings [61]. However, at a holding temperature of 1000 K, this trend diverged, with the order of the largest α average grain size being Fe-0.35 wt.%C, Fe-0.20 wt.%C, and Fe-0.50 wt.%C.

**Figure 19:**
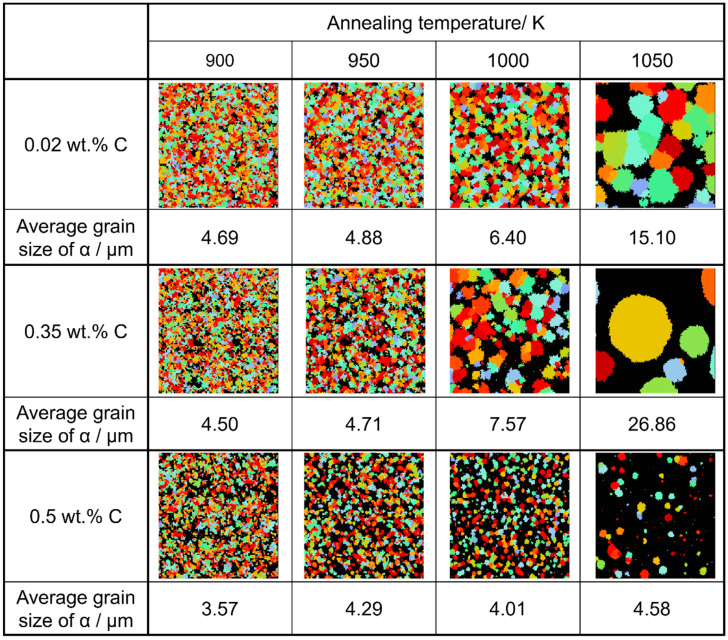
Relationship between carbon concentration, average grain size of α grain after the completion of phase transformation, and annealing temperatures in the simulated microstructures.

Considering the relationship between carbon concentration and the driving force for nucleation, it is evident that the lower the carbon concentration, the smaller the driving force for nucleation. Conversely, when considering the equilibrium volume fraction, the lower the carbon concentration added, the larger the α volume fraction observed. In the case of Fe-0.20 wt.%C, a substantial number of α grains of a specific size precipitate. However, in Fe-0.50 wt.%C, the driving force for nucleation is small, and the equilibrium α volume fraction is also low, resulting in fewer precipitated α grains and smaller grain sizes. Meanwhile, in Fe-0.35 wt.%C, although the driving force for nucleation decreases compared to Fe-0.20 wt.%C, the equilibrium α volume fraction remains substantial, allowing the α grains to grow sufficiently. Consequently, we can affirm that the relationship between carbon concentration and the average α grain size upon completion of the α phase transformation has been elucidated in Figure 19. In the temperature range below 950 K, the average α particle size decreases as the carbon content increases, consistent with previous findings. However, in the temperature range of 1000 K or higher, when comparing the results for 0.20 and 0.5 wt%C, a similar trend was observed. However, the carbon content in this case led to a structure that partially contradicted this trend.

**Figure 20:**
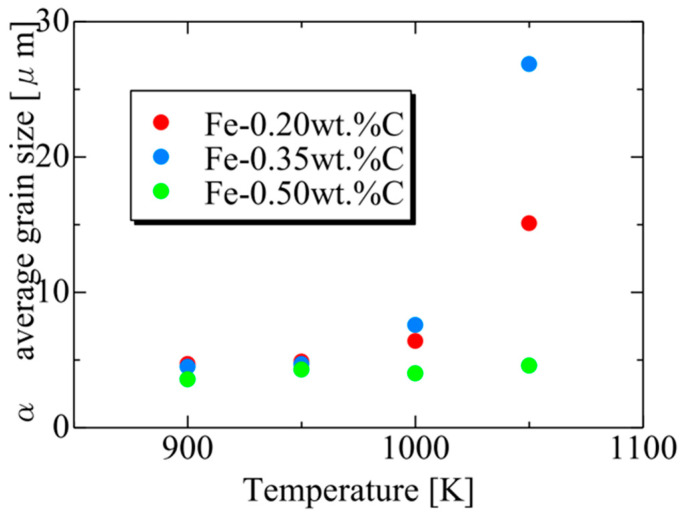
Relationship between the average α particle size and annealing temperature for each carbon concentration after the completion of phase transformation.

Figure 20 presents the relationship between the average α particle size and holding temperature for each carbon concentration after phase transformation. The results also capture the tendency for the average α grain size to increase upon the completion of the phase transformation due to enhanced carbon atom diffusion with increasing holding temperature. At each temperature, the average α grain size order upon completing α transformation is Fe-0.35 wt.%C, Fe-0.20 wt.%C, Fe-0.50 wt.%C. This order can also be attributed to the impact of the driving force for nucleation and the equilibrium volume fraction, as discussed previously [62].

## 4. Conclusions

By developing a cellular automaton model capable of replicating the phase transformation from austenite to ferrite, a typical behavior observed in carbon steel, and conducting Bayesian optimization of parameters for three different carbon concentrations, the following key findings were obtained:

1. The TTT diagrams depicting the onset of α phase transformation were predicted to align reasonably well with results from conventional experiments. Furthermore, it became evident that accounting for the incubation period of nucleation is crucial for accurate modeling.
2. In this nucleation incubation period-aware model, the incubation period constant was identified as a parameter significantly influenced by carbon concentration. The incubation period constant is mainly affected by carbon concentration and the optimized values have been obtained as 10^−24^, 10^−19^, and 10^−21^ corresponding to carbon concentrations of 0.2 wt%, 0.35 wt%, and 0.5 wt%, respectively. In contrast, other parameters displayed relatively minor dependencies on carbon concentration.
3. Realistic microstructure diagrams with appropriate grain sizes and shapes were successfully generated. It was observed that reducing the initial γ grain size decreased the average α grain size upon completing the α phase transformation.

These findings contribute to a better understanding of phase transformation behavior in carbon steel and underscore the importance of considering nucleation incubation periods in such models.

## Figures and Tables

**Table 1:** Summary of the parameters with optimal values.

Carbon Concentration	Incubation Period Constant	T_max_	dT_σ_
0.20 wt.%	10^−24^	850–900	40–75
	—	875–900	100–125
0.35 wt.%	10^−19^	875–900	40–60
	—	875–900	25–75
0.50 wt.%	10^−21^	825–875	40–75
	—	875–900	25–50

## Data Availability

Not applicable.
